# Beyond the embryo: reframing recurrent implantation failure through immunopriming and endometrial immune dysregulation

**DOI:** 10.3389/frph.2026.1892030

**Published:** 2026-10-01

**Authors:** Jayesh Amin

**Affiliations:** 1 Reproductive Medicine, Nova WINGS IVF, Ahmedabad, India; 2 Reproductive Genetics and Immunology, WINGS IVF Hospital, Ahmedabad, India

**Keywords:** embryo-endometrial crosstalk, endometrial receptivity, euploid blastocyst, immunopriming, recurrent implantation failure (RIF), reproductive immunology, uterine (u)NK cells

## Abstract

Recurrent implantation failure (RIF) remains one of the most debated and emotionally challenging conditions in assisted reproductive technology (ART). Despite remarkable advances in embryology, including blastocyst culture, time-lapse imaging and preimplantation genetic testing for aneuploidy (PGTA), a significant proportion of patients continue to fail implantation even after transfer of morphologically good-quality or euploid embryos. Contemporary reproductive medicine has largely adopted an embryocentric model of implantation, often defining RIF based upon repeated failed embryo transfers. However, this framework may represent a statistical oversimplification that inadequately reflects the biological complexity of implantation and the clinical realities faced by infertile couples. Emerging evidence increasingly supports the concept that implantation is not solely dependent on embryo competence but also requires synchronized endometrial receptivity and finely regulated maternal immune tolerance. The endometrium functions as an active immunological organ populated by uterine natural killer cells, macrophages, dendritic cells, cytokines, chemokines, and regulatory T cells, all of which contribute to embryo acceptance, trophoblast invasion, angiogenesis, and placentation. Dysregulation of this immune-endometrial dialogue may contribute substantially to implantation failure in selected patients. This perspective article critically examines the limitations of current RIF definitions, the overemphasis on embryo quality, and the under-recognized role of reproductive immunology. Furthermore, it explores the emerging concept of immunopriming and discusses potential immunological markers, peripheralendometrial immune crosstalk, individualized immune profiling, and immunomodulatory therapies such as intravenous immunoglobulin (IVIG) and lymphocyte immunotherapy (LIT). Although current evidence remains heterogeneous and definitive randomized controlled trials are lacking, outright dismissal of immune contributions to implantation failure may no longer be scientifically justified. We propose that implantation failure should be reconsidered as a multifactorial disorder involving embryo competence, endometrial receptivity, and immune equilibrium, thereby advocating for a more integrated and individualized approach to implantation medicine.

## Graphical Abstract

**Figure:**
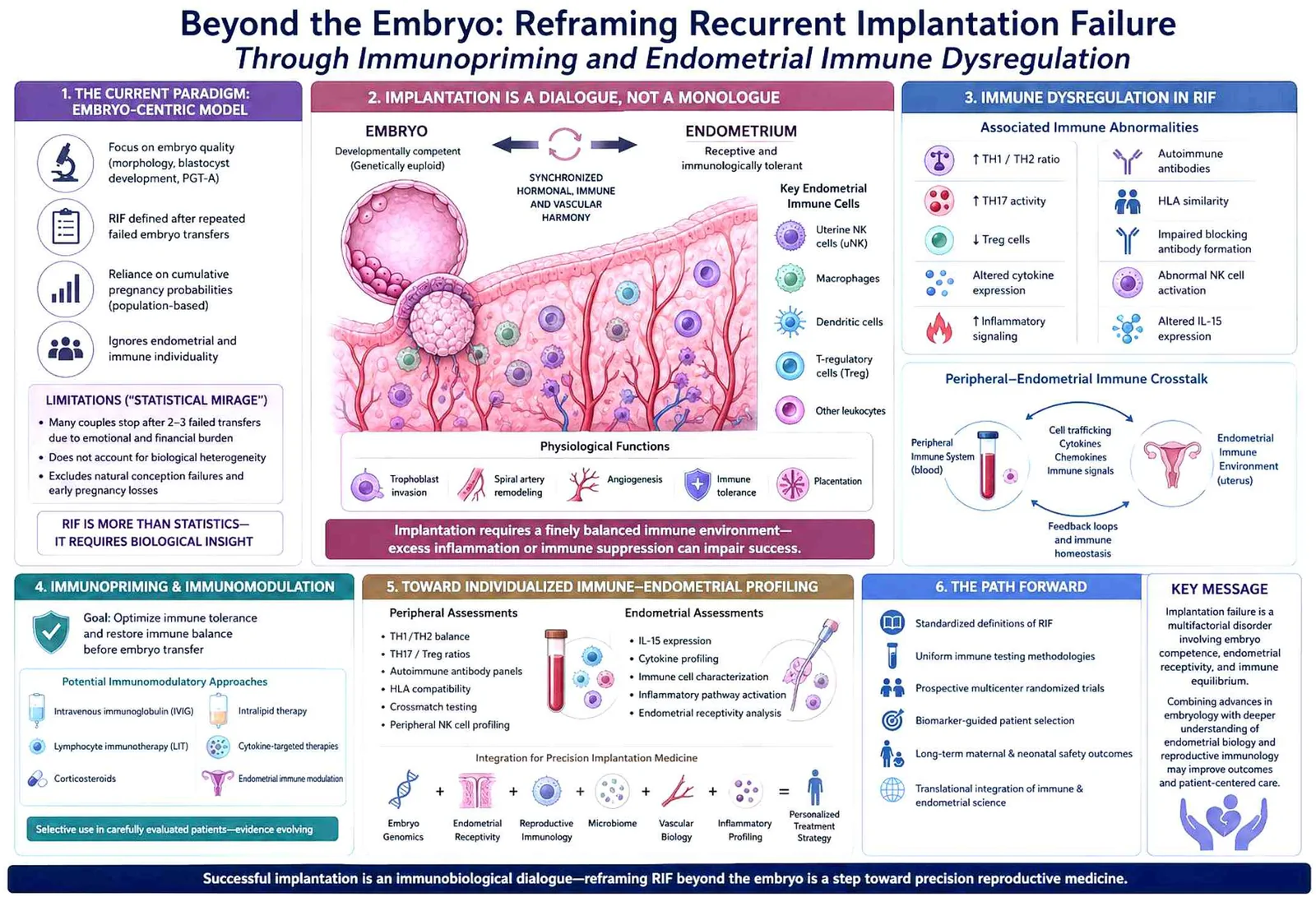

## Introduction

Human implantation represents one of the most complex biological events in reproductive medicine. Successful implantation requires precise synchronization between a developmentally competent embryo and a receptive endometrium within a tightly regulated hormonal, vascular and immunological environment (1). Over the past several decades, major technological advances in assisted reproductive technology (ART) have dramatically improved embryo culture systems, embryo selection strategies, and genetic screening approaches. Consequently, the embryo has increasingly become the focal point of implantation science. The evolution of ART reflects this progression. Early IVF strategies prioritized increasing the number of embryos transferred in order to improve pregnancy rates. Subsequently, attention shifted toward embryo morphology and blastocyst development. More recently, preimplantation genetic testing for aneuploidy (PGT-A) and euploid embryo transfer have become central components of modern IVF practice. This progression has strengthened the belief that implantation failure is primarily embryo-driven. However, an important clinical paradox persists. A substantial proportion of patients continue to experience repeated implantation failure despite transfer of morphologically optimal or genetically euploid embryos. These observations suggest that embryo competence alone cannot fully explain implantation outcomes (2). Increasing attention is therefore being directed toward the endometrium and the maternal immune environment. The emerging field of reproductive immunology challenges the traditional embryo-centric paradigm by proposing that implantation depends not only upon embryo quality but also upon immune-endometrial tolerance and maternal immunological adaptation (3). Yet despite growing interest in this area, reproductive immunology remains one of the most controversial fields in infertility medicine because of inconsistent evidence, heterogeneous methodologies, and lack of standardized clinical protocols (4). This perspective article examines the evolving concept of implantation failure through the lens of endometrial immune dysregulation and immunopriming. We explore the biological plausibility, clinical relevance, controversies, and future implications of integrating immune-endometrial assessment into implantation medicine.

### Recurrent implantation failure: the statistical mirage

One of the major challenges in reproductive medicine is the absence of a universally accepted definition for recurrent implantation failure (5). Various professional organizations define RIF differently based on the number of failed embryo transfers, the number of embryos transferred, maternal age, or embryo quality. Most definitions rely heavily on cumulative pregnancy probabilities following repeated ART attempts. From a statistical perspective, cumulative pregnancy rates continue to improve after multiple embryo transfers in patients with diminished ovarian reserve (6) Consequently, some guidelines argue that several failed transfers may still fall within expected statistical variability rather than representing a pathological implantation disorder. While statistically reasonable, this interpretation may inadequately reflect clinical reality. In real-world practice, many couples are unable to continue repeated IVF cycles indefinitely (7). Emotional burnout, financial burden, treatment fatigue, and psychological stress frequently lead patients to discontinue treatment after two or three failed transfers. Therefore, the concept that implantation failure should only be recognized after multiple failed cycles may delay investigation into potentially correctable biological abnormalities. This creates what may be described as a “statistical mirage,” wherein population-based cumulative success rates obscure the biological individuality of patients experiencing repeated failure.

The 2022 Lugano Workshop consensus highlighted the uncertainty surrounding the diagnosis of recurrent implantation failure (RIF), characterizing it as a potential “statistical mirage” and estimating that true RIF may occur in fewer than 5% of couples undergoing ART. The consensus emphasized that RIF has been overdiagnosed and overtreated and recommended reassurance and continuation of conventional management for most patients following ART failure. Importantly, however, the consensus also acknowledged that a biologic basis for repeated implantation failure may exist in a small subset of patients and that investigation may be justified after repeated failure, depending on the quality and cost of the diagnostic test and the efficacy of the associated treatment. Thus, the Lugano position does not support routine immune testing, but rather underscores the need to distinguish patients with sporadic ART failure from the smaller subgroup in whom persistent biologic mechanisms may warrant investigation. In this context, endometrial immune profiling should currently be regarded as an investigational approach requiring rigorous validation rather than as a routine clinical test. Future studies should determine whether biologically defined subgroups of patients can be identified reproducibly and whether immune-profile-guided interventions improve reproductive outcomes in appropriately selected patients (8).

Our perspective is that the clinical and biological significance of repeated implantation failure should be considered alongside, rather than solely within, statistical models of cumulative IVF success. A patient experiencing three failed euploid embryo transfers may not perceive reassurance from statistical models predicting eventual pregnancy after five or six transfers. Instead, such repeated failures may warrant consideration of underlying defects in endometrial receptivity or maternal immune tolerance. Moreover, existing definitions frequently exclude patients who experience repeated biochemical pregnancies, unexplained infertility, or recurrent early pregnancy losses outside ART settings. Such exclusion may further limit our understanding of implantation as a broader physiological phenomenon rather than a process confined to IVF laboratories.

Thus, the limitations of current RIF definitions highlight the need for more biologically informed approaches that integrate embryological, endometrial, vascular, and immunological factors rather than relying solely upon statistical expectations.

### Implantation beyond the embryo

The remarkable progress achieved in embryology has undoubtedly transformed reproductive medicine. However, the increasing focus on embryo genetics and morphology has unintentionally contributed to relative neglect of the endometrium (9). Implantation is fundamentally a bidirectional interaction between embryo and endometrium and thus a competent embryo alone may be insufficient if the maternal endometrium is not receptive and immunologically tolerant. The embryo itself represents a semi-allogenic structure containing paternal antigens foreign to the maternal immune system (10). Successful implantation therefore requires carefully regulated maternal immune adaptation. Excessive inflammatory activation may impair trophoblast invasion, whereas excessive immune suppression may interfere with implantation signaling and placental development (11).

The endometrium is not merely a passive surface for embryo attachment. It is a dynamic immunological organ that undergoes cyclical remodeling and contains a highly specialized immune microenvironment (12). During the implantation window, the endometrium becomes populated with uterine natural killer (uNK) cells, macrophages, dendritic cells, T-regulatory cells, cytokines, and chemokines that collectively orchestrate implantation. Approximately 70% of decidual leukocytes consist of uterine NK cells (13) (Figure 1).

**Figure 1:**
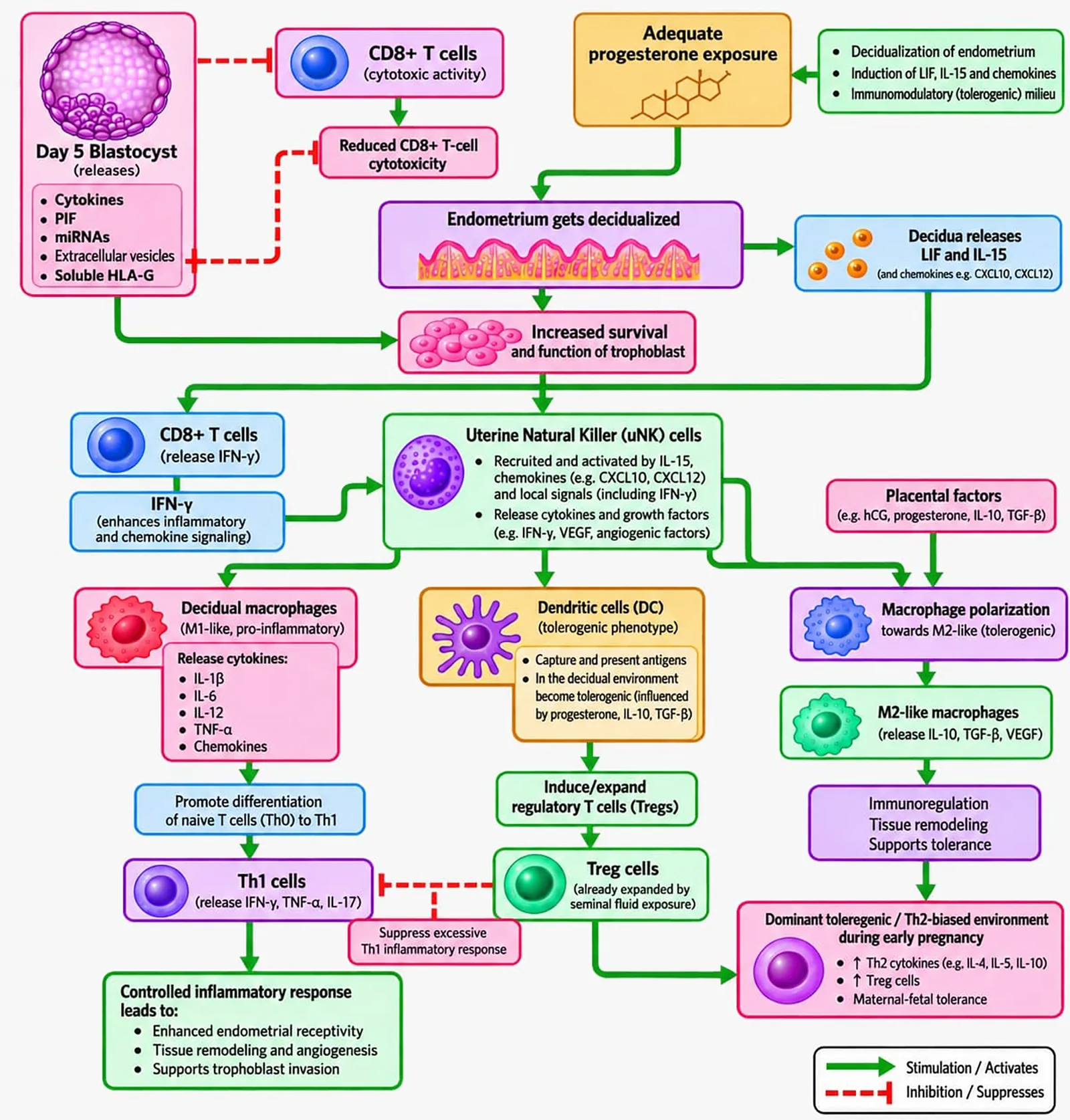
Immune–endometrial cross-talk required for successful implantation. This figure illustrates the key cellular and molecular interactions between the blastocyst, decidualized endometrium, trophoblast, and maternal immune system that contribute to successful implantation. Progesterone and embryo/placental-derived signals promote endometrial decidualization, trophoblast survival and function, controlled inflammatory responses, tissue remodeling, angiogenesis, and trophoblast invasion. Concurrent activation of regulatory immune pathways, including Treg cells and tolerogenic macrophage and dendritic-cell responses, limits excessive inflammation and promotes maternal–fetal immune tolerance, thereby supporting successful implantation and early pregnancy. CD8+, cluster of differentiation 8–positive; IFN-*γ*, interferon-gamma; TNF-α, tumor necrosis factor-alpha; PIF, preimplantation factor; miRNA, microRNA; HLA-G, human leukocyte antigen-G; LIF, leukemia inhibitory factor; IL, interleukin; CXCL10, C-X-C motif chemokine ligand 10; CXCL12, C-X-C motif chemokine ligand 12; uNK, uterine natural killer; NK, natural killer; VEGF, vascular endothelial growth factor; M1, classically activated/pro-inflammatory macrophage phenotype; DC, dendritic cell; TGF-*β*, transforming growth factor-beta; Th0, T-helper 0; Th1, T-helper 1; Th2, T-helper 2; Treg, regulatory T cell; M2, alternatively activated/tolerogenic or tissue-remodeling macrophage phenotype; hCG, human chorionic gonadotropin.

Unlike peripheral NK cells, uNK cells are not primarily cytotoxic. Instead, they play essential physiological roles in trophoblast invasion, spiral artery remodeling, angiogenesis, placental development, and maternal-fetal immune tolerance (14). Persistent implantation failure despite transfer of euploid embryos strongly suggests that embryo competence alone cannot explain all failed implantations. Increasing evidence supports the possibility that defective endometrial immune regulation may contribute significantly to implantation failure in selected patients.

### Peripheral-endometrial immune crosstalk

One of the most controversial topics in reproductive immunology involves the relationship between peripheral immune markers and uterine immune function. Several guidelines question the clinical relevance of peripheral blood NK cell testing because peripheral NK cells may not directly correlate with uterine NK populations (15). However, emerging evidence suggests that peripheral immune cells may still contribute to endometrial immune homeostasis and replenishment. Although the correlation between peripheral and uterine immune profiles remains imperfect, complete dismissal of peripheral immune assessment may be overly reductionist. In many other medical specialties, immune-mediated disorders are diagnosed and treated despite incomplete mechanistic understanding. Autoimmune thyroiditis, inflammatory bowel disease, autoimmune encephalitis, and rheumatologic disorders all involve complex immune interactions that continue to evolve scientifically. Yet clinicians routinely incorporate immunological assessment into clinical decision-making in these cases but when it comes to reproductive medicine, the scenario is quite different. In reproductive medicine, the immune contribution to implantation failure often remains viewed with skepticism. Part of this skepticism stems from historical overuse of empirical immunological therapies without robust scientific validation. Nevertheless, growing translational research increasingly supports the biological plausibility of immune involvement in implantation.

Several immunological abnormalities have been associated with implantation failure including Elevated TH1/TH2 ratios, Increased TH17 activity, Reduced T-regulatory cell populations, Altered cytokine expression, Increased inflammatory signaling, Autoimmune antibodies, HLA similarity between couples, Impaired blocking antibody formation, Abnormal NK cell activation and Altered IL-15 expression. Although none of these markers individually demonstrate sufficient predictive accuracy, however when combined immune profiling is done, it may provide clinically meaningful inference (16).

### Immunopriming and reproductive immunomodulation

The concept of immunopriming has emerged as a potential strategy to optimize maternal immune tolerance before embryo transfer. It aims to modulate the maternal immune environment in a manner that promotes embryo acceptance while avoiding excessive inflammatory or cytotoxic responses. The rationale behind immunopriming is biologically plausible. Implantation requires a controlled inflammatory response during the initial stages followed by immune tolerance for ongoing pregnancy maintenance. Dysregulation of this balance may impair implantation.

Potential immunomodulatory approaches include Intravenous immunoglobulin (IVIG), Lymphocyte immunotherapy (LIT), Corticosteroids, Intralipid therapy, Cytokine-targeted therapies and Endometrial immune modulation. Among these approaches, IVIG and LIT remain particularly controversial. Critics emphasize the lack of large randomized controlled trials and concerns regarding cost, safety, and inconsistent outcomes (17). However, several clinicians continue utilizing these therapies selectively in highly individualized clinical scenarios, particularly in patients with repeated implantation failure associated with abnormal immune profiles. Interestingly, current guidelines often adopt a cautious rather than dismissive stance. Some organizations discourage routine use of IVIG because of insufficient evidence and potential complications, yet simultaneously acknowledge possible benefit in selected “high-risk” or “red flag” patients. This ambiguity reflects ongoing scientific uncertainty rather than definitive disproval (18). The existence of selective recommendations suggests that the issue may not be absence of efficacy, but rather insufficient evidence to define which patients are most likely to benefit. This highlights the importance of developing standardized immunological testing and biomarker-guided treatment strategies rather than universal empirical therapy (19).

### Toward individualized immune-endometrial profiling

The future of implantation medicine may depend upon integration of embryo assessment with individualized endometrial and immune profiling. Potential peripheral assessments include TH1/TH2 balance, TH17/Treg ratios, Autoimmune antibody panels, HLA compatibility, Crossmatch testing and Peripheral NK cell profiling. Potential endometrial assessments include IL-15 expression, Cytokine analysis, Endometrial immune cell characterization, Inflammatory pathway activation and Endometrial receptivity analysis (20). Importantly, no single biomarker currently demonstrates sufficient sensitivity or specificity for universal application. Also, implantation failure itself is unlikely to represent a single disease entity. Rather, it may reflect multiple overlapping biological pathways. Combining immune profiling with embryological assessment may therefore enable identification of clinically relevant implantation phenotypes. Such an approach could help to identify patients requiring deeper immune evaluation, avoid unnecessary immunotherapy exposure, improve patient selection and facilitate individualized treatment protocols.

## Discussion

Current definitions of recurrent implantation failure remain inconsistent and controversial. Most of the professional societies define RIF only after repeated failed embryo transfers in ART cycles. Such definitions are largely based upon cumulative statistical probabilities rather than individualized biological variations. This approach creates multiple limitations. Many patients discontinue treatment after only two or three failed cycles because of financial exhaustion, emotional trauma, and psychological distress. Furthermore, restricting implantation failure exclusively to ART settings ignores the possibility that similar implantation dysfunction may exist during natural conception cycles also. Simon and Laufer emphasized that recurrent implantation failure (RIF) remains a poorly defined clinical condition, with no universally accepted diagnostic criteria. The authors proposed that RIF should be considered after failure of implantation in at least three consecutive IVF cycles despite the transfer of one or two high-quality embryos in each cycle. They highlighted that successful implantation depends on a complex interaction between a competent embryo and a receptive endometrium, and that defects in either component may lead to repeated implantation failure. The review concluded that RIF is a multifactorial condition involving embryonic, uterine, genetic, thrombophilic, immunological and male factors, and therefore requires a comprehensive and individualized diagnostic approach. Importantly, the authors stressed that no single factor can explain all cases of RIF and that evaluation should focus on identifying potentially treatable abnormalities in both partners. They further suggested that management should be directed toward correcting specific underlying causes rather than relying on empiric treatments, because the evidence supporting many interventions remains limited. Overall, the article established RIF as a complex syndrome resulting from impaired embryo–endometrial interaction and highlighted the need for personalized investigation and treatment strategies rather than a uniform therapeutic approach (21). Margalioth and colleagues defined RIF as the failure to achieve pregnancy after at least three IVF cycles in which good-quality embryos were transferred. The authors emphasized that repeated implantation failure should be distinguished from sporadic implantation failure because repeated unsuccessful transfers despite apparently adequate embryos suggest the presence of underlying pathological factors beyond chance alone. However, the definition did not incorporate maternal age, embryo ploidy, or cumulative implantation probability. The major limitation of this definition is the assumption that all transferred embryos possess equivalent developmental competence. This study highlighted the need for systematic investigation of uterine, embryonic, and immunological factors in women experiencing repeated IVF failure (22). Coughlan et al. provided one of the most influential definitions of RIF, describing it as the failure to achieve a clinical pregnancy after transfer of at least four good-quality embryos in a minimum of three fresh or frozen cycles in women younger than 40 years. Importantly, this definition introduced embryo quality and maternal age into the diagnostic criteria. The authors argued that implantation failure should be interpreted relative to the expected implantation potential of the transferred embryos rather than simply the number of failed cycles. Their review also proposed a multifactorial model involving embryonic, endometrial, anatomical, genetic, thrombophilic, and immunological contributors (23). In a large international survey involving clinicians and embryologists, Cimadomo et al. demonstrated striking global heterogeneity in the diagnosis and management of RIF. Definitions varied from two failed embryo transfers to multiple failed cycles. The study showed that even among reproductive specialists there is no consensus regarding when implantation failure becomes pathological. The authors concluded that the lack of standardization impairs both clinical decision-making and the comparability of research studies (24). The evolution of RIF definitions clearly demonstrates a progressive transition from simplistic cycle-based definitions toward individualized prognostic models. Early definitions focused primarily on the number of failed IVF attempts or transferred embryos, whereas later definitions incorporated embryo quality, maternal age, and embryo ploidy. The international survey by Cimadomo et al. confirmed substantial variability in clinical practice, while Polanski et al. highlighted that this heterogeneity undermines the comparability of research studies and to improve uniformity, they proposed defining RIF as failure to achieve implantation after two consecutive IVF/ICSI or frozen embryo transfer cycles involving the transfer of at least four good-quality cleavage-stage embryos or two good-quality blastocysts (25). More recent frameworks proposed by Ata et al. (26), Rozen et al. (27), and ultimately ESHRE 2023 (28) recognize that implantation probability is patient-specific and influenced by embryo competence and reproductive prognosis. Collectively, these studies suggest that RIF should not be considered a single uniform clinical entity but rather a heterogeneous syndrome encompassing multiple embryonic, endometrial, immunological, genetic, and environmental pathways that culminate in a common phenotype of repeated implantation failure.

Nathalie Lédée and colleagues (29) pioneered the concept of endometrial immune profiling, proposing that implantation depends not merely on the quantity of uterine natural killer (uNK) cells but also on their functional maturation and cytokine balance, assessed using biomarkers such as IL-15/Fn-14 and IL-18/TWEAK. In a prospective observational cohort of 394 women with unexplained recurrent implantation failure, personalized management based on endometrial immune profiling was followed by a live birth rate of 39.8% at the first subsequent embryo transfer. However, the observational, non-randomized design, together with the absence of a randomized untreated comparator, precludes causal inference regarding the benefit of immune-profile-guided treatment. Subsequent systematic reviews have confirmed that women with recurrent implantation failure generally exhibit higher endometrial uNK levels than controls but have also demonstrated that peripheral NK-cell measurements do not reliably reflect uterine immune status, elevated uNK levels do not consistently predict live birth, and substantial methodological heterogeneity limits reproducibility. Collectively, these findings suggest that while endometrial immune profiling represents a promising research tool, further multicenter validation, methodological harmonization, and assay standardization are required before routine clinical implementation can be recommended.

Woon et al. (30) conducted a comprehensive systematic review and meta-analysis evaluating both the number and functional characteristics of uterine natural killer (uNK) cells in women with recurrent miscarriage (RM) and recurrent implantation failure (RIF). The authors reported that women with RIF generally exhibited significantly higher endometrial uNK-cell densities compared with fertile controls, suggesting an association between altered uterine immune profiles and implantation failure. However, the review also demonstrated that peripheral blood NK-cell measurements showed poor correlation with uterine NK-cell populations, indicating that peripheral immune assessments cannot reliably serve as surrogates for the endometrial immune microenvironment. Importantly, despite the observed increase in uNK-cell numbers, elevated uNK-cell density alone was not consistently associated with subsequent live birth or reproductive outcomes. Furthermore, substantial heterogeneity was identified across the included studies with respect to patient selection, definitions of RIF, timing of endometrial biopsy, laboratory techniques, immunohistochemical protocols, functional assays, and threshold values used to define abnormal uNK-cell levels. The authors therefore concluded that, although uterine immune dysregulation is biologically plausible and may contribute to implantation failure in selected patients, the current evidence does not support routine clinical use of uNK-cell testing until standardized methodologies, validated reference ranges, and adequately powered prospective studies establish its diagnostic and prognostic utility.

In a retrospective case–control study, Tohma et al. (31) evaluated NK-cell subsets in both peripheral blood and uterine lavage samples in women with unexplained recurrent implantation failure (RIF). The authors found that peripheral blood NK-cell analysis did not reliably differentiate women with RIF from infertile controls, highlighting its limited diagnostic utility. In contrast, assessment of uterine immune-cell subsets demonstrated significant differences in uterine NK (uNK) cell populations, suggesting that the local endometrial immune environment may be more informative than systemic immune markers. The authors concluded that peripheral NK-cell testing should not be routinely used to investigate RIF and proposed that uterine immune assessment may represent a more biologically relevant approach, although further validation in larger prospective studies is required before its clinical implementation.

Supporting the cautious interpretation, Woon et al. (32) performed a systematic review and meta-analysis specifically evaluating immunotherapy in women with recurrent implantation failure (RIF) or recurrent miscarriage (RM) selected on the basis of abnormal natural killer (NK) cell levels or activity. Unlike previous reviews, this study focused on biomarker-selected patients and assessed the efficacy of intravenous immunoglobulin (IVIG), intralipid, prednisolone, and etanercept. The pooled analysis demonstrated a significantly higher live birth rate following IVIG therapy (RR 2.57, 95% CI 1.79–3.69), while individual randomized controlled trials also reported improved reproductive outcomes with etanercept, intralipid, and prednisolone, with statistically significant benefits observed for etanercept and intralipid. Nevertheless, the authors emphasized that these encouraging findings were derived from a limited number of studies with considerable clinical heterogeneity, moderate-to-high risk of bias, and variability in NK-cell assessment methods and treatment protocols. Consequently, they concluded that the available evidence remains insufficient to support the routine clinical use of immunotherapy, even in women selected based on abnormal NK-cell profiles. Importantly, this recommendation reflects the limited quality and standardization of the current evidence rather than proven ineffectiveness of these interventions. The authors advocated for large, well-designed randomized controlled trials using scientifically validated immunological biomarkers and standardized patient selection criteria to determine the true clinical benefit of immunotherapy in women with unexplained reproductive failure.

Moustakli et al. (33) comprehensively reviewed the immunological mechanisms underlying recurrent pregnancy loss (RPL) following IVF and highlighted the pivotal roles of regulatory T cells, uterine natural killer (uNK) cells, cytokine dysregulation, and impaired maternal–fetal immune tolerance in reproductive failure. The authors also discussed emerging immunomodulatory therapies and the potential of immune profiling to facilitate precision medicine. However, they emphasized that significant controversies remain regarding the diagnostic validity of immune biomarkers and the clinical effectiveness of interventions such as intralipid, intravenous immunoglobulin, corticosteroids, and anticoagulants. They concluded that the marked heterogeneity of immune phenotypes and the lack of robust prospective evidence necessitate standardized immunological assessment and adequately powered clinical trials before precision immunotherapy can be routinely integrated into fertility practice.

Yang et al. (34) demonstrated that recurrent implantation failure (RIF) is not a uniform clinical entity but comprises at least two distinct endometrial molecular subtypes: an immune-driven subtype (RIF-I) characterized by activation of inflammatory and immune pathways, and a metabolic-driven subtype (RIF-M) associated with altered oxidative metabolism and steroid biosynthesis. Their machine-learning classifier accurately distinguished these subtypes and identified potential subtype-specific therapeutic targets. These findings reinforce the concept that the pathogenesis of RIF is biologically heterogeneous and suggest that future management should move beyond a “one-size-fits-all” approach toward biomarker-guided precision medicine. However, prospective clinical validation is required before molecular subclassification can be routinely incorporated into clinical practice.

Importantly, acknowledging immune contributions to implantation failure does not imply rejection of embryology. Embryo quality unquestionably remains one of the most important determinants of pregnancy success. However, persistent failure despite transfer of competent embryos strongly suggests that additional biological mechanisms contribute in selected patients (35). The field of reproductive immunology occupies a uniquely controversial position within fertility medicine. On one hand, increasing biological and translational evidence supports the role of immune-endometrial interactions in implantation. On the other hand, insufficient standardization and lack of robust randomized trials continue to limit widespread clinical acceptance. The challenge moving forward is to avoid both extremes; one is indiscriminate overuse of unvalidated immunotherapies and another is complete dismissal of reproductive immunology despite emerging evidence. Future research must prioritize standardized definitions of RIF, Uniform immune testing methodologies, Prospective multicenter randomized trials, Biomarker-guided patient selection, Translational integration of immune and endometrial science and Long-term maternal and neonatal safety outcomes.

Equally important is recognition of the emotional burden experienced by patients with repeated implantation failure (36). Statistical reassurance regarding cumulative pregnancy rates may not adequately address the psychological trauma associated with repeated failed implantation. A more individualized and biologically informed approach may therefore improve not only clinical outcomes but also patient-centered care. Ultimately, successful implantation may best be understood as an immunobiological dialogue rather than a purely embryological event. The future of implantation medicine will likely require integration rather than polarization; combining advances in embryology with deeper understanding of endometrial biology and reproductive immunology.

Our perspective is that the current lack of high-quality evidence supporting routine immune-based diagnostic or therapeutic interventions should not be conflated with definitive evidence that the underlying biological mechanisms are absent or that targeted interventions are ineffective. Various professional and working guideline groups have appropriately considered many such investigations experimental or insufficiently validated, reflecting the limited, heterogeneous, and predominantly observational nature of the available evidence. In this context, the way forward may not be either indiscriminate immune testing or complete dismissal of immunological mechanisms, but rather the rigorous identification of biologically plausible patient subgroups followed by adequately powered, prospective, randomized controlled trials. Such studies are needed to determine whether targeted immune evaluation or intervention provides meaningful clinical benefit and to distinguish potentially useful individualized approaches from unnecessary blanket interventions.

## Conclusion

Recurrent implantation failure is increasingly recognized as a heterogeneous condition in which embryonic competence, endometrial receptivity, endocrine regulation, vascular remodeling, genetics, and maternal immune adaptation interact in a complex and dynamic manner. Although accumulating evidence supports a biologically plausible role for reproductive immunology in selected patients, the current evidence is insufficient to support the routine use of immune biomarkers or immunomodulatory therapies in clinical practice. Importantly, the cautious position adopted by major professional societies should not be interpreted as evidence of therapeutic ineffectiveness, but rather as a reflection of the limited availability of high-quality, standardized, and reproducible clinical evidence. We believe that the future of reproductive immunology lies not in abandoning this field, but in strengthening it through standardized immune phenotyping, validated biomarkers, and adequately powered multicentre randomized controlled trials. Such collaborative research will help identify the patients most likely to benefit from targeted immunomodulation and enable the development of robust evidence-based recommendations. Ultimately, unexplained recurrent implantation failure should increasingly be viewed as an underexplored rather than an inexplicable condition, with continued scientific investigation offering the greatest opportunity to translate promising biological insights into safe, personalized, and effective clinical care.

## Data Availability

The original contributions presented in the study are included in the article/Supplementary Material, further inquiries can be directed to the corresponding author.
